# Birt-Hogg-Dubé syndrome: a large single family cohort

**DOI:** 10.1186/s12931-016-0339-2

**Published:** 2016-02-29

**Authors:** Kate Skolnik, Willis H. Tsai, Kimberly Dornan, Renée Perrier, Paul W. Burrowes, Warren J. Davidson

**Affiliations:** Department of Medicine, University of Calgary, Calgary, AB Canada; Department of Community Health Sciences, University of Calgary, Calgary, AB Canada; Department of Medical Genetics, University of Calgary, Calgary, AB Canada; Department of Diagnostic Imaging, University of Calgary, Calgary, AB Canada

**Keywords:** Cystic lung disease, Birt-Hogg- Dubé syndrome, Pneumothorax

## Abstract

**Background:**

Birt-Hogg-Dubé (BHD) syndrome is an autosomal dominant condition characterized by dermatologic lesions, pulmonary manifestations, and renal tumors. The syndrome arises from germline mutations in the folliculin *(FLCN)* gene*.* We present findings from the single largest family BHD cohort described to date. Primary objectives were to characterize cystic lung changes on computed tomography (CT) chest scanning and identify features that stratify patients at higher risk of pneumothorax. Secondary objectives entailed description of the following: type and natural history of BHD-associated pneumothorax, pulmonary function characteristics, and relationship between cystic lung changes and pulmonary function.

**Methods:**

The study was a retrospective chart review for a case series of a single family. Over 70 family members of a proband with documented BHD were identified, 68 of which consented to genetic testing. All those with confirmed BHD were offered a clinical assessment by the Medical Genetics and Pulmonary services which included a history, physical exam, complete pulmonary function tests, and computed tomography (CT) scan of the chest and abdomen.

**Results:**

Thirty-six individuals had a heterozygous mutation in the *FLCN* gene (c.59delT). Of these, 100 % (28/28) had pulmonary cysts, 41 % (13/32) had spontaneous pneumothoraces, 26 % (8/31) had kidney cysts, 3 % (1/31) had renal tumors, and 53 % (18/34) had dermatologic manifestations. Recurrent pneumothoraces were common (40 %). Cyst size (OR 3.23, 95 % CI 1.35–7.73) and extent of lower lung zone disease (OR 6.43, 95 % CI 1.41–29.2) were the only findings associated with pneumothorax. The size or extent of cystic disease did not correlate with lung function results.

**Conclusions:**

This is the largest single family cohort of patients with BHD syndrome documented to date. We found that all individuals had pulmonary cysts, pneumothoraces were common, and cyst size and lower lobe predominant disease were associated with pneumothorax. Lung function was generally preserved and not affected by a high cyst burden.

## Background

Birt-Hogg- Dubé (BHD) syndrome is a rare autosomal dominant condition characterized by skin lesions, pulmonary cysts, spontaneous pneumothoraces, and renal tumors [1].

It was first described as an inherited dermatologic syndrome in the 1970s, however future studies identified pulmonary and renal manifestations as key features of the disease [2]. The gene responsible for the syndrome, *FLCN,* was mapped to chromosome 17p12q11.2 [3] and cloned in 2002 [4]. The product, folliculin, is found in many normal tissues and thought to play a role in tumor suppression [4]. There are over 70 unique pathogenic *FLCN* gene mutations [5].

BHD dermatologic lesions are common and include fibrofolliculomas (face, trunk, and arm papules), trichodiscomas, and acrochordons (skin tags) [6]. Renal cysts and tumors (including chromophobe renal cell carcinoma) are frequently seen and are often multiple and bilateral [7]. Lung cysts are also common and the age-adjusted risk of pneumothorax has been estimated as high as 50.3 [8, 9].

Only 430 families worldwide have been documented with BHD Syndrome [10, 11], with the largest reporting twenty-five affected members [12]. We present findings from the largest single family cohort described to date. The primary objective of the case series was to characterize the cystic lung changes on computed tomography (CT) chest scanning and identify features that stratify patients at higher risk of pneumothorax. The secondary objectives included: (1) description of the type and natural history of BHD-associated pneumothorax; (2) pulmonary function characteristics; and (3) influence of the degree of cystic lung changes on pulmonary function.

## Methods

### Design

The study was a retrospective chart review for a case series of a single family. Genetic testing, pulmonary function tests, and imaging were performed as part of the patients’ routine clinical assessment. Our study did not proceed to formal ethics review given the retrospective and case series nature of the project.

### Population

The proband was diagnosed with BHD in the context of several unprovoked pneumothoraces and a strong family history of spontaneous pneumothoraces. A heterozygous mutation in the *FLCN* gene (c.59delT), resulting in premature protein truncation, was identified in this individual. Following the diagnosis of BHD syndrome, cascade genetic testing was offered to family members with possible clinical features of BHD and on a predictive basis for asymptomatic at-risk individuals,with 68 people agreeing to formal genetic testing. Inclusion criteria for the case series included: (1) age 18 and greater (2) direct relation to the proband (not through marriage) and (3) documentation of the *FLCN* gene mutation.

**Fig. 1:**
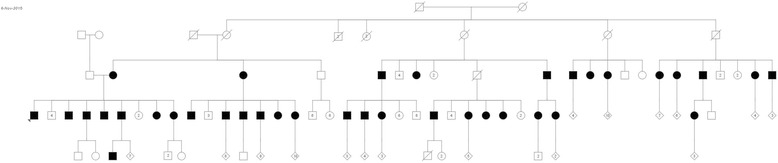
Pedigree of BHD affected family. The proband is indicated by the arrow on the far left. BHD affected individuals are highlighted in black

A family history was compiled using information provided by the proband and multiple relatives. The pedigree included over 150 individuals from six generations originating from a matriarch and patriarch born in the late nineteenth century (Fig. 1). The proband was one of 13 siblings.

### Genetic assessment and confirmation of BHD syndrome

Genetic testing was performed using the same standardized assay and equipment in the Alberta Children’s Hospital Molecular Diagnostics Laboratory. Initial sequencing on the proband was performed following Polymerase Chain Reaction (PCR) amplification using primers flanking each exon allowing for analysis of exonic plus flanking intronic sequence. Subtraction analysis using Mutation Surveyor (SoftGenetics) software was used to identify differences between the patient and a reference sequence. The Molecular Diagnostic Lab then performed targeted sequence analysis to detect this mutation in consenting family members. Genomic DNA isolated from whole blood was PCR amplified for exon 4 of the *FLCN* gene and sequenced using the Sanger method to identify the presence of the *FLCN* c.59delT mutation.

### Clinical assessment

All individuals with the *FLCN* mutation were offered a clinical assessment by a respirologist including history, physical exam, CT scan of the chest, CT or magnetic resonance imaging (MRI) scan of the abdomen, and complete pulmonary function tests. Lung function testing, including spirometry and diffusing capacity for carbon monoxide (DLCO), was performed in accordance with ATS standards and using the well-validated Knudson 1983 normset [13–15]. Lung function tests performed at the time of the individual’s BHD diagnosis were used for data analysis. Clinical data was not collected from family members who did not have the gene mutation or undergo genetic testing.

### Chest imaging

Non-contrast, chest CT with 5 mm slices were performed. Images were reviewed by a thoracic radiologist who was blinded to the clinical history. CT scans were scored according to the following criteria: (1) cyst size (based on greatest diameter on axial sections), (2) cyst number (1, 2 to 5, or numerous), (3) extent of upper lung zone involvement (0–5 %, 5–25 %, 25–50 %, 50–75 %, or greater than 75 % of area affected by cysts), (4) extent of lower lung zone involvement (0–5 %, 5–25 %, 25–50 %, 50–75 %, or greater than 75 % of area affected by cysts), and (5) cyst distribution (upper lung zone predominant, lower lung zone predominant, right side predominant, left side predominant, or equal through-out). The lungs were divided into upper and lower zones at the level of the right main pulmonary artery. There is no existing rating system for cystic lung disease. Therefore, the divisions for extent of upper and lower lung involvement as well as cyst number were arbitrary and made a priori.

### Statistical analysis

Descriptive analysis was applied to the patient characteristics. Simple logistic regression was performed to identify variables associated with pneumothorax. A full model was constructed from variables identified as associated with pneumothorax on univariate analysis (*p* <0.05). Backwards selection was performed by successively dropping the least significant variables until all remaining variables were statistically significant (Stata STAT/IC 13 software).

## Results

### Cohort demographic and clinical characteristics

**Fig. 2:**
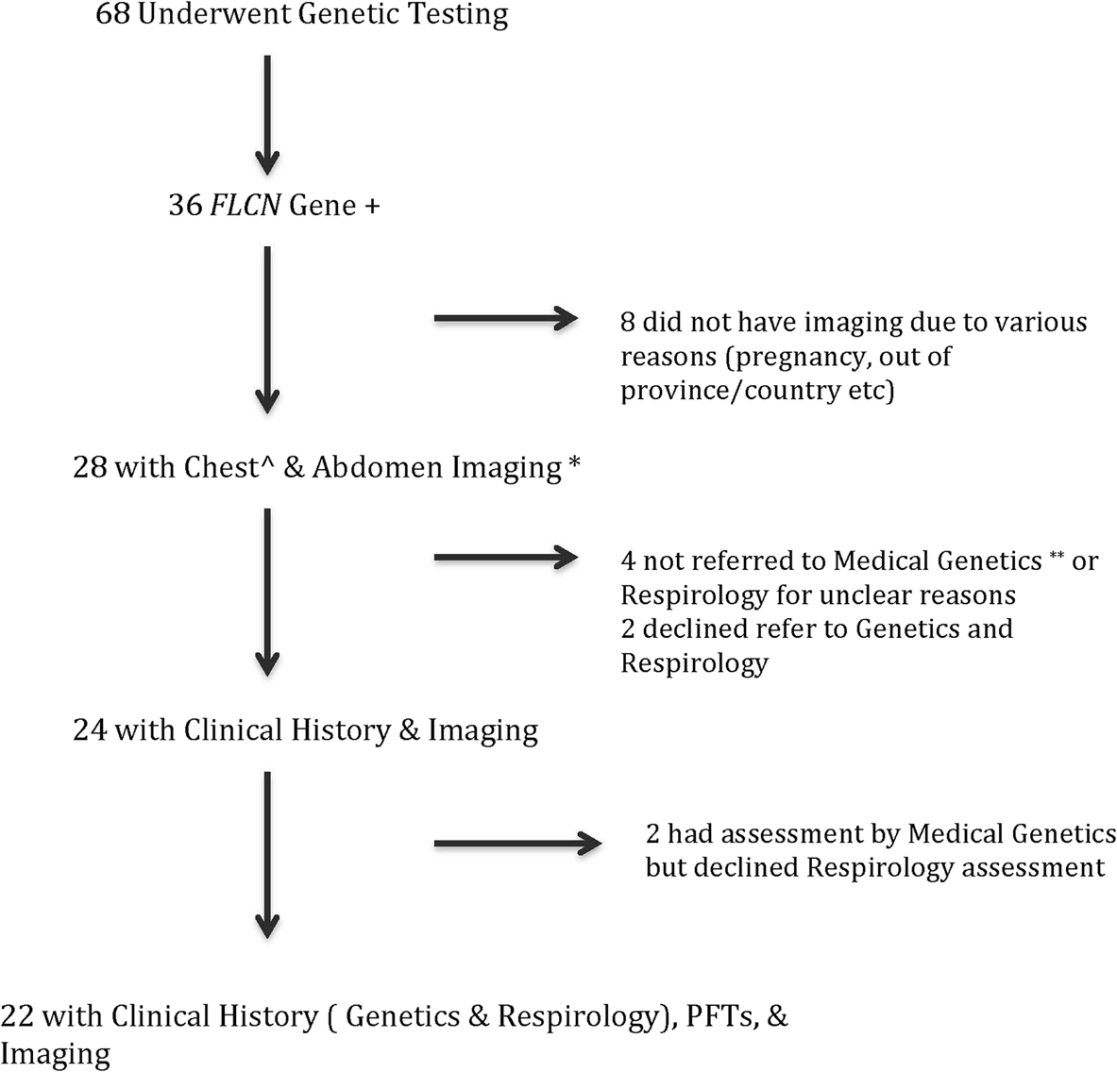
Patient Flow Chart. ^Chest imaging entailed a CT scan. *Abdominal imaging imaging was performed with MRI in 3 individuals (due to radiation or contrast concerns with CT). ** Referral to Medical Genetics refers to a formal appointment to review patient clinical history (genetic testing may have been performed in individuals due to family history with their consent in the absence of formal appointment Medical Genetics appointment with functional inquiry)

Over seventy individuals from a single family were identified as biologic relatives of the proband and 68 of these consented to genetic testing. Of those tested, 36 were positive for the *FLCN* c.59delT. Twenty-eight individuals had CT scans of the chest and abdomen and 22 of these had a complete clinical assessment (Fig. 2).

Baseline characteristics of the study population are summarized in Table 1. There were similar numbers of men and women. The average age at diagnosis was 42 years (range of 19–71 years). More than 50 % of subjects had an unknown smoking status or were current or former smokers. Seventeen percent of the group had asthma (as defined in Table 1). Thirty-six percent (8/22) had airflow obstruction; 6 were secondary to asthma and 2 due to unknown etiology. Of those who had diffusing capacity for carbon monoxide (DLCO) testing, the majority (11/19 or 58 %) were within normal limits. Seven subjects (37 %) had an elevated DLCO.

**Table 1:** Baseline characteristics

Characteristic	Value
Age at Diagnosis, yrs	42 (3.1)
Male:Female	18:18
BMI, kg/m^2^	25.7 (0.85)
Smokers, n (%)	
Current	3 (9)
Former	4 (11)
Never	17 (47)
Unknown status	12 (33)
Smoking, pack years	6.9 (3.05)
FEV_1_, L	3.32 (0.21)
FEV_1_ (% pred)	92 (3.64)
FVC, L	4.40 (0.27)
FVC (% pred)	101 (4.10)
FEV_1_/FVC	74.8 (2.07)
DL_CO_, mL/min/mmHg	33.32 (2.26)
DL_CO_ (% pred)	130 (6.72)
DL_CO_/VA, mL/min/mmHg	27.1 (3.66)
DL_CO_/VA (% pred)	130 (5.67)
DL_CO_, mL/min/mmHg (Asthmatics excluded)	33.0 (2.56)
DL_CO_ (% pred) (Asthmatics excluded)	133.9 (7.40)
Asthma diagnosis, n (%)	6 (17)

Asthma was defined as full reversibility of airflow obstruction following bronchodilator administration or a positive methacholine challenge test. Data are shown as mean (SD)

*Abbreviations*: *BMI* body mass index, *DL*_*CO*_ diffusing capacity for carbon monoxide, *FEV1* forced expiratory volume in one second, *FRC* functional residual capacity, *FVC* forced vital capacity

### Manifestations of BHD syndrome

Clinical sequelae of BHD syndrome are summarized in Table 2. All patients who underwent CT chest imaging had pulmonary cysts. The mean cyst size was 2.9 cm (SD 0.37) ranging from 0.7 to 9 cm in maximal diameter. The majority (83 %) had lower lung zone predominant cysts. There was no association between smoking and cyst size or number. Thirteen subjects (41 %) had at least one spontaneous pneumothorax. The mean age at first pneumothorax was 35.6 (SD 2.37**)** years. Seven subjects had multiple pneumothoraces; one individual had bilateral involvement at presentation. Among the remaining 6 people who experienced recurrent episodes, half of them had 4 pneumothoraces each. Of the 25 total pneumothoraces, there were a similar number of right and left sided occurrences (13 and 11, respectively; the location of one was unknown). Ten of the pneumothoraces were recurrent, 6 of which were on the contralateral side.

**Table 2:** Clinical features of bhd cohort

Finding	Prevalence (%)
Pulmonary	
Cystic Lesions	28/28 (100)
Pneumothorax	13/32 (41)
Dermatological	18/34 (53)
Fibrofolliculomas	18/34 (53)
Trichodiscomas	0/34 (0)
Acrochordons	2/34 (6)
Renal	
Cystic Lesions	8/31 (26)
Malignant Lesions	1/31 (3)
Other Malignancy	1/31 (3)

Of the total pneumothorax episodes, 21 (84 %) underwent an interventional procedure; chest tube insertion in 9 cases and thoracic surgery (as primary treatment) in 12. Of those undergoing surgical intervention, 8 were treated with bullectomy/pleurectomy, 2 underwent combination pleurectomy and wedge resection, 1 was managed with lobectomy alone, and in 1 case the surgical details were unclear. The number of pleurodesis procedures or the type (chemical or physical) could not be ascertained from the available data. The recurrence rate for pneumothoraces managed by chest tube alone was 16 % (1/6) and for those managed by surgical intervention was 9 % (1/11).

Dermatologic manifestations (primarily fibrofolliculomas) occurred in 53 % of the cohort. Twenty-six percent had kidney cysts and one individual was diagnosed with an unclassified renal cell carcinoma on routine screening, which was successfully removed. One individual had a pituitary tumor, a variant of spindle cell oncocytoma. No direct correlation between BHD syndrome and pituitary tumors has been documented [6].

There were no clinical features or measures of pulmonary function associated with higher likelihood of pneumothorax (Table 3). In contrast, both cyst size (OR 3.23, 95 % CI 1.35–7.73) and extent of lower lung zone disease (OR 6.43, 95 % CI 1.41–29.2) were associated with pneumothorax. Specifically, those with pulmonary cysts with a maximum diameter less than 2.9 cm had a significantly lower risk of spontaneous pneumothorax (OR 0.06, 95 % CI 0.01–0.40). Individuals with more than 25 % lower lung zone cyst involvement were 5 times more likely to experience pneumothorax (OR 4.57, 95 % CI 0.88–23.7). Other imaging features, including cyst number, cyst distribution, and extent of upper lung zone disease were not associated with a higher likelihood of pneumothorax (Table 3).

**Table 3:** Pneumothorax risk factors

Baseline characteristics	OR	SD	P	95 % CI
Sex	0.40	0.30	0.23	0.09–1.76
Age	0.97	0.03	0.43	0.92–1.03
BMI	1.03	0.11	0.79	0.83–1.28
Height	0.97	0.05	0.67	0.88–1.07
Smoker	1.00	-	-	-
Smoking Pack years	1.18	0.14	0.15	0.94–1.47
Asthma	0.35	0.35	0.29	0.05–2.47
FEV_1_/FVC absolute	0.92	0.05	0.16	0.83–1.03
FEV_1_ % predicted	0.98	0.026	0.52	0.93–1.03
FVC % predicted	1.00	0.02	0.88	0.96–1.05
DLCO % predicted	0.98	0.017	0.24	0.95–1.01
Renal Cysts	1.33	1.23	0.75	0.22–8.22
Dermatologic findings	1.30	0.94	0.72	0.32–5.33
Cyst Number				
Few (<5 cysts)	0.51	0.47	0.47	0.08–3.14
Numerous (>5)	1.96	1.82	0.47	0.32–12.1
Cyst Size	3.23	1.44	0.008	1.35–7.73
0–2.9 cm diameter	0.06	0.06	0.003	0.01–0.40
3–10 cm diameter	15.4	14.3	0.003	2.49–95.1
Cyst Distribution	0.62	0.22	0.18	0.30–1.25
Extent of Upper Lung	1.75	1.12	0.38	0.50–6.15
Zone Disease (% volume affected by cysts)				
< 25 % of UL	2.25	2.74	0.51	0.21–24.4
> 25 % of UL	0.44	0.54	0.51	0.04–4.82
Extent of Lower Lung	6.43	4.96	0.016	1.41–29.2
Zone Disease (% volume affected by cysts)				
< 25 % of LL	0.25	0.22	0.12	0.04–1.45
> 25 % of LL	4.57	3.89	0.07	0.88–23.7

Asthma was defined as full reversibility of airflow obstruction following bronchodilator administration or a positive methacholine challenge test

*Abbreviations*: *BMI* body mass index, *DLC*_*O*_ diffusing capacity for carbon monoxide, *FEV*_*1*_ forced expiratory volume in one second, *FVC* forced vital capacity, *UL* upper lobe, *LL* lower lobe

## Discussion

This is the largest single family BHD cohort reported in the literature to date. There are approximately 430 families with BHD syndrome documented in the literature. We found that all individuals had pulmonary cysts, pneumothoraces were common, and cyst size and lower lobe predominant disease were associated with pneumothorax. Lung function was generally preserved and not affected by a high cyst burden.

Previous studies included multiple families and were characterized by several genotypes [1, 8, 9, 12, 16–24]. In contrast, our cohort consisted of a single Caucasian family with a *FLCN* c.59delT mutation; thus providing a more homogenous phenotype.

In our cohort, every individual had pulmonary cysts on CT chest imaging. The high penetrance of lung cysts was in keeping with existing BHD literature (83 to 100 %) [9, 12, 16–21, 23, 24]. The prevalence of pneumothorax was slightly higher than that observed in the four largest BHD studies, which ranged from 24 to 32 % [1, 8, 9, 12]. However, pneumothorax prevalence as low as 8 % [22, 24] and as high as 93 % [20] has been described. Cyst size and extent of lower lung zone disease were found to be associated with pneumothorax. This is supported by previous literature, although cyst volume, number, and right middle location have also been reported to be associated with pneumothorax risk [9]. In contrast to Zbar and colleagues’ finding [8], age was not associated with pneumothorax.

Our recurrent pneumothorax rate was 42 %, which falls between the previously reported rates of 25 and 75 % [8, 9]. Most of the documented pneumothoraces required management with an interventional procedure, similar to prior studies [9]. The recurrence rates were substantially reduced post treatment, especially post surgery. Furthermore, it has been suggested that chemical pleurodesis or pleural ablation (which can be used to prevent recurrence of primary and secondary pneumothoraces) would be of even greater benefit in BHD given the often diffuse nature of the lung disease [25]. Others have extrapolated from lymphangioleiomyomatosis (LAM) data that chemical pleurodesis and surgical intervention have comparable outcomes in preventing pneumothorax recurrence [26].

The optimal method and timing of treating BHD-associated pneumothorax is unclear and controversies exist as randomized prospective trials are lacking. Some reviews suggest that BHD-associated pneumothorax should be managed the same as in the general population [27]; others argue that with the lower likelihood of spontaneous resolution and the high likelihood of recurrence, pneumothoraces should be managed more aggressively in this population [26]. Given the available data [9] in combination with our findings we would suggest the latter; clinicians should have a lower threshold to treat BHD-associated pneumothorax with tube thoracostomy (where indicated) as well as consider early referral for a surgical opinion.

Our study had the largest collection of BHD subjects to date who underwent lung function testing. Interestingly, we found the majority had preserved DLCO, with a subset having supranormal values. This was not explained by test technique, equipment problems, body surface area, or asthma exacerbation. Although the reason for these outliers is unclear, this finding could possibly be explained by factors that were not systematically assessed (such as polycythemia, early heart failure, pulmonary hemorrhage, pretest exercise, or the occurrence of a Mueller maneuver during testing). In contrast, prior studies documented a low normal or mildly reduced DLCO in the context of BHD [17, 18, 28]. Lung volumes were preserved in our cohort, consistent with existing literature [17, 18, 28]. Our study suggests that individuals with BHD may have even milder impairments in lung function that previously thought [17, 18]. A novel finding was the observation that burden of cystic lung disease did not affect lung function.

With respect to extrapulmonary manifestations, dermatological findings were noted in only 53 % of the cohort, which was lower than the 79–100 % prevalence of skin involvement documented in most BHD cohorts [1, 9, 12, 24]. This could be related to the relatively young average age at diagnosis in this cohort, as BHD related skin findings are more common starting in the third to fourth decade of life [27]. Renal cysts were found in 26 % of those with imaging, which was higher than the 15 % observed in the only other study that specifically quantified renal cysts [24]. In contrast, the 3 % prevalence of renal tumors documented in our cohort was significantly lower than the 12–27 % seen in the four largest BHD studies [1, 8, 9, 12]. This may be explained by sample size as some smaller cohorts also quoted 5 to 6 % prevalence of renal cancer [20, 22].

There are a few limitations to this study. First, this is a retrospective review. However, the clinical assessments were very detailed with pulmonary function testing and imaging performed in most subjects. Second, the sample size is smaller than previously reported pooled cohorts. However, this is the largest single-family cohort described in the literature with the same genotype, allowing analysis of a uniquely homogenous population. Furthermore, our study highlights the concept that understanding the clinical phenotype of an individual with BHD might predict the phenotype of other affected family members and aid in personalizing risk estimates and tailoring management.

Although rare, BHD can have significant clinical implications. There are no well-validated studies of BHD prevalence [29], but some sources estimate 9 per 1 million [30]. Of note, up to 5 % of apparent primary spontaneous pneumothorax have been attributed to underlying BHD [31]. This highlights the importance of inquiring about family history, skin abnormalities, and renal disease in individuals with pneumothorax. It is also important to recognize that individuals with BHD cystic lung disease may be underdiagnosed since lung function is generally well preserved (in contrast to conditions such as LAM).

## Conclusion

We describe the largest single family cohort of patients with BHD syndrome reported to date. All subjects had cystic lung disease with a significant number experiencing spontaneous pneumothorax. Cyst size and lower lung zone predominant disease were associated with pneumothorax occurrence. Lung function was generally preserved and not affected by a high cyst burden. Given that BHD-associated pneumothoraces may be severe and recurrent, clinicians may consider early intervention and thoracic surgery referral.

## Abbreviations

BHD: Birt-Hogg-Dubé syndrome
CT: Computed tomography
MRI: Magnetic resonance imaging
OR: Odds ratio
